# Elevated blood urea nitrogen is associated with recurrence of post-operative chronic subdural hematoma

**DOI:** 10.1186/s12883-020-01985-w

**Published:** 2020-11-10

**Authors:** Ning Wang, Jiangnan Hu, Anthony Oppong-Gyebi, Xuanhao Zhu, Yihao Li, Jianjing Yang, Linhui Ruan, Qichuan Zhuge, Sheng Ye

**Affiliations:** 1 Zhejiang Provincial Key Laboratory of Aging and Neurological Disorder Research, The First Affiliated Hospital of Wenzhou Medical University, Wenzhou, 325000 China; 2 Department of Neurosurgery, The First Affiliated Hospital of Wenzhou Medical University, Wenzhou, 325000 Zhejiang China; 3 Department of Pharmacology and Neuroscience, University of North Texas Health Science Center, Fort Worth, TX 76107 USA

**Keywords:** Chronic subdural hematoma, Blood urea nitrogen (BUN), Recurrence rate

## Abstract

**Background:**

Chronic subdural hematoma (CSDH) is fundamentally treatable with about a 2–31% recurrence rate. Recently, there has been renewed interest in the association between Blood Urea Nitrogen (BUN) and intracranial lesion. Therefore, this paper attempts to show the relationship between BUN and CSDH recurrence.

**Methods:**

A total of 653 CSDH cases with Burr-hole Irrigation (BHI) were enrolled from December 2014 to April 2019. The analyzed parameters included age, gender, comorbidities, laboratory investigations, medication use and hematoma location. The cases were divided into recurrence and non-recurrence groups while postoperative BUN concentration was further separated into quartiles (Q1 ≤ 4.0 mmol/L, 4.0 < Q2 ≤ 4.9 mmol/L, 4.9 < Q3 ≤ 6.4 mmol/L, Q4 > 6.4 mmol/L). Restricted cubic spline regressions and logistic regression models were performed to estimate the effect of BUN on CSDH recurrence.

**Results:**

CSDH recurrence was observed in 96 (14.7%) cases. Significant distinctions were found between recurrence and non-recurrence groups in postoperative BUN quartiles of cases (*P* = 0.003). After adjusting for the potential confounders, the odds ratio of recurrence was 3.069 (95%CI =1.488–6.330, *p* = 0.002) for the highest quartile of BUN compared with the lowest quartile. In multiple-adjusted spline regression, a high BUN level visually showed a significantly high OR value of recurrence risk.

**Conclusions:**

Elevated BUN at post-operation is significantly associated with the recurrence of CSDH, and it is indicated that high levels of serum BUN after evacuation may serve as a risk factor for CSDH recurrence.

**Supplementary Information:**

The online version contains supplementary material available at 10.1186/s12883-020-01985-w.

## Background

Chronic subdural hematoma (CSDH) is a frequently encountered neurosurgical disorder that is common among the aged. The incidence of CSDH is estimated to be 8.2–17.6 per 100,000 persons per year which increases along with increasing age [1–3]. Burr-hole irrigation (BHI) is most widely used to treat CSDH with a relatively good outcome, but about 2–31% of patients relapse after the initial operation [1].

Factors found to influence CSDH recurrence have been reported in several studies, including age, Glasgow coma score (GCS), antiplatelet or anticoagulant agents, bilateral hematomas, postoperative pneumocephalus, and other certain related computed tomography (CT) findings [4–7]. However, the findings of the connection between laboratory investigation and CSDH recurrence are still scarce.

Blood urea nitrogen (BUN) concentration is an easily overlooked investigation. Interestingly, there is a growing amount of literature that recognizes high BUN/creatinine(Cr) as an independent risk factor of poor outcomes in acute ischemic stroke (AIS) patients [8–10]. Furthermore, elevated BUN concentration was revealed to increase in-hospital mortality in AIS patients [11]. These studies indicated that BUN was associated with intracranial disorders. But very little attention has been paid to the relationship between BUN and CSDH recurrence so far.

In this paper, variables associated with CSDH recurrence were investigated, focusing on the postoperative BUN concentrations of patients with CSDH who underwent evacuation at a single facility from 2014 to 2019. This research aimed to explore the relationship between serum BUN levels and CSDH recurrence to provide a new orientation to study the pathophysiology of CSDH.

## Methods

### Patients and parameters

This study enrolled admitted patients with the diagnosis of CSDH at the First Affiliated Hospital of Wenzhou Medical University, Zhejiang province, China, between December 2014 and April 2019. Patients with CSDH were diagnosed by head magnetic resonance imaging (MRI) and CT. The following exclusive criteria were used:(1) without surgical treatment;(2) younger than 18 years old;(3) severe epilepsy;(4) severe renal or blood diseases;(5) hematoma organized or bad operation result;(6) craniotomy or evacuated by other departments;(7) lack of laboratory investigation;(8) hospital mortality. Patient with two operations was defined as two cases.

The following clinical characteristics were analyzed: age, gender, demographic parameters, comorbidities, postoperative laboratory investigations, postoperative medication use, location of hematoma, and preoperative BUN level. Glasgow outcome scale (GOS) at discharge was performed to evaluate the neurologic function of patients.

### Surgery procedure and follow-up

All patients underwent BHI with general anesthesia. After burr-hole exposure, dura incision and irrigation, we used closed system drainage with a subdural catheter connected to a vacuum plastic bag. Most catheters were withdrawn within 72 h after the operation. Head CT was performed within the first 48 h and on day 6 or 7 postoperatively. For good measure, patients were followed up with head CT at 3 months at the outpatient service.

### Definition of recurrence and grouping

**Fig. 1:**
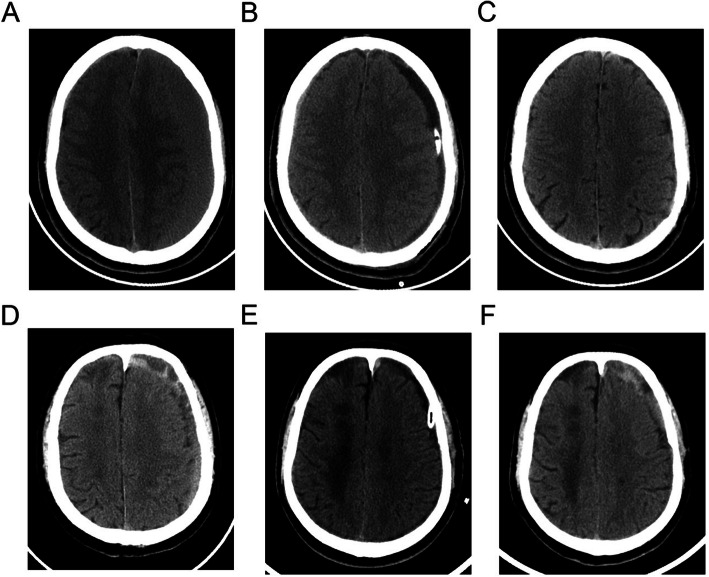
CT imagings of two typical patients without and with recurrence of CSDH. **a**, the preoperative presentation of patient A without recurrence; **b** the imaging of the same patient within 48 h postoperatively; **c** the CT scan at 3-month follow-up of the patient A; **d** the presurgical imaging of patient B with recurrence of CSDH; **e** the presentation of patient B within 48 h postoperatively; **f** the CT scan of patient B at 2 months after operation, a significant increase volume of subdural collection could be seen in left side

CSDH recurrence was evaluated by radiological criteria, which included an increased volume of the subdural collection and brain compression on either side within 3 months compared with those first measured after surgery through CT scans, and clinical criteria in which preoperative symptoms and signs abided or recurred (Fig. 1). The classification of recurrence was determined by two experienced neurosurgeons who were blinded to the study.

The cases were first divided into 2 groups according to recurrence and non-recurrence to investigate the risk factors for CSDH recurrence. To identify the specific effect of postoperative BUN, it was further divided into quartiles to explore the different influences on the CSDH recurrence in each category. The local ethics committee ruled that no formal ethics approval was required in this particular study.

### Statistical analysis

Data for continuous variables were shown as mean ± standard deviation or medians and interquartile range. To verify whether the data follows a normal distribution or not, Kolmogorov-Smirnov test was performed. Categorical variables were shown as relative frequencies and percentages. Normally distributed variables were compared by Student’s t-test, whereas the Mann–Whitney U test was used for the asymmetrically distributed continuous variables. Chi-squared (χ^2^) test was used to compare categorical variables. Statistical comparisons among BUN concentrations stratification were estimated by one-way analysis of variance (ANOVA) or Kruskal–Wallis test for continuous variables, and Pearson’s chi-square test for categorical variables where appropriate. Wilcoxon signed-rank test was performed to compare the difference between pre and post-operative BUN levels. Odds ratio (OR) and 95% confidence interval (CI) for recurrence risk were calculated by performing multivariate-adjusted binary logistic regression. Restricted cubic spline regressions model was built to examine the linear relation between serum BUN concentration and the risk of recurrence. In this study, a two-tailed *p*-value less than 0.05 (*P* < 0.05) was considered as statistical significance. All statistical analyses were done using SPSS (version 23.0, IBM Corp.) and STATA software (version 12, StataCorp LP) was employed to analyze the restricted cubic spline regressions.

## Results

### Baseline characteristics of all cases in the recurrence group and non-recurrence group

**Fig. 2:**
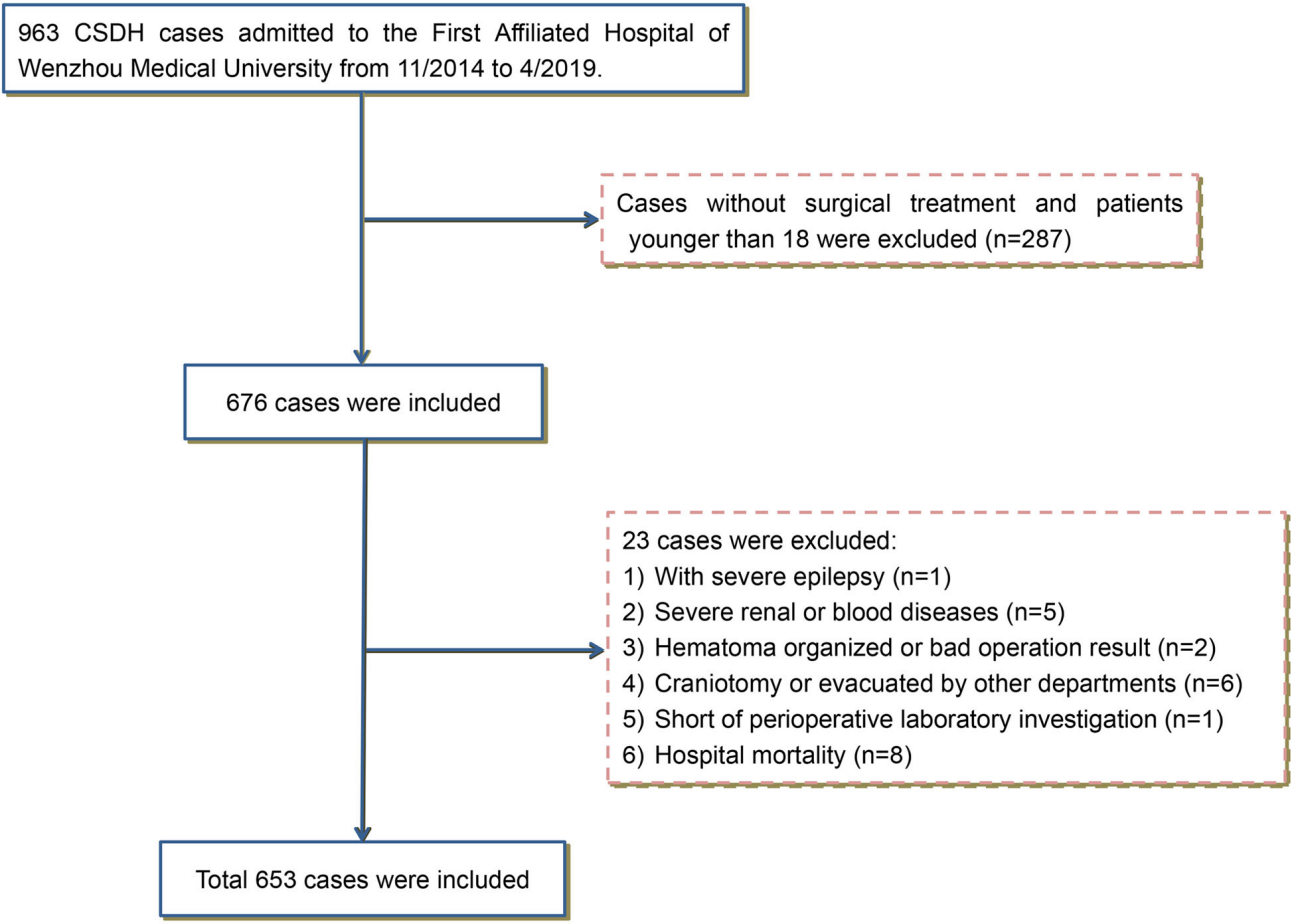
Study flow diagram. CSDH, chronic subdural hematoma

A total of 653 CSDH cases were enrolled in this study (Fig. 2). This study group included 561 male cases (85.9%) and 92 female cases (14.1%). Patient’s age ranged from 21 to 100 years with a median of 72 years (interquartile range 64 to 80 years). (Table 1). 8 patients with hospitalized mortality were excluded and there was no mortality upon follow-up in this study.

**Table 1:** Clinical and Demographic Characteristics of Patients with CSDH

Variables	All patients (*N* = 653)	Recurrence(*n* = 96)	Non-Recurrence(*n* = 557)	*P*-value
Postoperative BUN (mmol/L)	4.9 (4,6.4)	5.9 (4.4,6.9)	4.9 (3.9,6.3)	< 0.001
**Demographic parameters**				
Age (years)	72 (64,80)	75 (69,82)	71 (63,80)	0.012
Gender				0.263
Male, n (%)	561 (85.9%)	86 (89.6%)	475 (85.3%)	
Female, n (%)	92 (14.1%)	10 (10.4%)	82 (14.7%)	
Current drinking, n (%)	234 (35.8%)	34 (35.4%)	200 (35.9%)	0.926
Current smoking, n (%)	237 (36.3%)	33 (34.4%)	204 (36.6%)	0.672
Baseline SBP (mmHg)	138 (126,153)	135 (124,151)	140 (127,153)	0.122
Baseline DBP (mmHg)	79 (71,87)	77 (71,84)	79 (71,87)	0.091
**Laboratory investigation**				
Blood glucose (mmol/L)	5.5 (4.7,6.6)	5.7 (5,6.7)	5.4 (4.7,6.6)	0.265
Cr (μmol/L)	67 (58,77)	68 (60,80)	66 (57,77)	0.097
Leukocyte (X10^9/L)	6.94 (5.81,8.43)	6.51 (5.52,7.55)	7.01 (5.85,8.62)	0.010
Neutrophil (X10^9/L)	4.62 (3.68,6.15)	4.30 (3.51,5.31)	4.70 (3.71,6.26)	0.012
Lymphocyte (X10^9/L)	1.46 (1.10,1.81)	1.46 (1.17,1.83)	1.47 (1.1,1.81)	0.650
Erythrocyte (X10^12/L)	4.23 ± 0.51	4.19 ± 0.52	4.24 ± 0.51	0.379
Hemoglobin (g/L)	132 (120,142)	132 (119,142)	132 (119,140)	0.442
Platelet (X10^9/L)	211 (172,249)	194 (164,239)	213 (175,251)	0.010
Prothrombin Time(s)	13.3 (12.8,13.9)	13.4 (12.8,14.0)	13.3 (12.8,13.9)	0.642
INR	1.02 (0.97,1.08)	1.02 (0.98,1.10)	1.02 (0.97,1.07)	0.719
Fibrinogen (g/L)	3.61 (3.09,4.28)	3.50 (2.89,4.15)	3.64 (3.15,4.30)	0.046
**Comorbidities**				
Hypertension, n (%)	252 (38.6%)	40 (41.7%)	212 (38.1%)	0.503
Diabetes mellitus, n (%)	77 (11.8%)	11 (11.5%)	66 (11.8%)	0.913
Cardiovascular disease, n (%)	42 (6.4%)	6 (6.3%)	36 (6.5%)	0.937
**Medication use**				
Atorvastatin therapy, n (%)	478 (73.2%)	74 (77.1%)	404 (72.5%)	0.352
PAMBA therapy, n (%)	189 (28.9%)	26 (27.1%)	163 (29.3%)	0.663
**Hematoma location**				0.957
Unilateral left, n (%)	288 (44.1%)	42 (43.8%)	246 (44.2%)	
Unilateral right, n (%)	216 (33.1%)	31 (32.3%)	185 (33.2%)	
Bilateral, n (%)	149 (22.8%)	23 (24.0%)	126 (22.6%)	
**Postoperative bleeding**	136 (20.8%)	22 (22.9%)	114 (20.5%)	0.585
**GOS at discharge**				0.549
5, n (%)	593 (90.8%)	90 (93.8%)	503 (90.3%)	
4, n (%)	48 (7.4%)	5 (5.2%)	43 (7.7%)	
3, n (%)	12 (1.8%)	1 (1.0%)	11 (2.0%)	
**Preoperative investigation**	*N* = 632	*n* = 95	*n* = 537	
BUN (mmol/L)	5.3 (4.4,6.5)	5.9 (4.8,6.9)	5.2 (4.3,6.4)	0.001

**NOTE.** SBP, systolic blood pressure; DBP, diastolic blood pressure; Cr, creatinine; INR, international normalized ratio; PAMBA, para-aminomethylbenzoic acid; BUN, blood urea nitrogen; GOS, Glasgow Outcome Scale

The descriptive characteristics between groups with and without recurrence are shown in Table 1, including the demographics, laboratory, imaging, medication and comorbidity characteristics. In this study, 96 (14.7%) cases were diagnosed as CSDH recurrence, including 16 patients who needed a second operation. Compared with non-recurrence, the cases in the group of recurrence were older and have lower levels of serum leukocyte, neutrophil and platelet count. However, lower serum fibrinogen concentration was examined in recurrence cases (*P* < 0.05). Moreover, there was a statistical difference between pre and post-operative groups on serum BUN concentration (*P* = 0.001 and *P* < 0.001, respectively). Table 2 revealed that the BUN level of the pre-operative group was significantly higher than the post-operative BUN level in the non-recurrence group (*P* < 0.001) while it showed no obvious difference in the recurrence group.

**Table 2:** The comparison between pre- and post-operative BUN levels

Variable	Preoperative BUN (mmol/L)	Postoperative BUN (mmol/L)	*P*-value
Total (n = 632)	5.3 (4.4,6.5)	4.9 (4.0,6.4)	< 0.001
Recurrence (n = 95)	5.9 (4.8,6.9)	5.85 (4.4,6.9)	0.177
Non-Recurrence (n = 537)	5.2 (44.3,6.4)	4.9 (3.9,6.25)	< 0.001

BUN, blood urea nitrogen;

### Baseline characteristics of all cases in BUN quartiles

For further exploration, the cases were divided into 4 groups based on quartiles of the postoperative BUN concentration. The cut-off points for this stratification of the BUN concentration into quartiles were: Q1 ≤ 4.0 mmol/L, 4.0 < Q2 ≤ 4.9 mmol/L, 4.9 < Q3 ≤ 6.4 mmol/L and Q4 > 6.4 mmol/L. Table 3 summarizes the characteristics of the CSDH cases by the quartiles of BUN. Cases with different postoperative BUN concentration appeared to be similar in most features except for age, Cr, erythrocyte and hemoglobin. These factors would be adjusted for multivariate-adjusted binary logistic regression for good measure. 22 (14%) of 157 patients in the highest quartile of BUN suffered moderate disability at discharge which was statistically higher than in other quartiles.

**Table 3:** Baseline Characteristics of Patients with Chronic Subdural Hematoma according to postoperative BUN quartile

Variables	BUN quartiles				
	quartile 1 *n* = 170(≤4.0)	quartile 2 *n* = 162(> 4.0,≤4.9)	quartile 3 *n* = 164(> 4.9,≤6.4)	quartile 4 *n* = 157(> 6.4)	*P*-value
BUN (mmol/L)	3.4 (3.0,3.7)	4.5 (4.3,4.8)	5.7 (5.3,6.0)	7.4 (6.9,8.3)	< 0.001
**Demographic parameters**					
Age (years)	68 (61,76)	72 (63,79)	73 (66,82)	76 (69,83)	< 0.001
Gender, male, n (%)	140 (82.4%)	148 (91.4%)	141 (86.0%)	132 (84.1%)	0.103
Current drinking, n (%)	61 (35.9%)	62 (38.3%)	58 (35.4%)	53 (33.8%)	0.866
Current smoking, n (%)	68 (39.4%)	56 (34.6%)	63 (38.4%)	51 (32.5%)	0.526
Baseline SBP (mmHg)	138.4 ± 19.4	139.3 ± 19.7	141.0 ± 20.6	142.5 ± 21.0	0.274
Baseline DBP (mmHg)	79 (71,87)	79 (73,86)	77 (72,88)	78 (71,87)	0.984
**Laboratory investigation**					
Blood glucose (mmol/L)	5.5 (4.8,6.5)	5.6 (4.7,6.6)	5.6 (4.7,6.8)	5.4 (4.5,6.7)	0.818
Cr (μmol/L)	60 (54,68)	66 (57,75)	69 (60,80)	74 (63,87)	< 0.001
Leukocyte (X10^9/L)	7.00 (6.14,8.45)	6.97 (5.81,8.62)	6.98 (5.73,8.33)	6.77 (5.55,8.39)	0.677
Neutrophil (X10^9/L)	4.78 (3.84,6.31)	4.68 (3.76,6.30)	4.52 (3.59,5.92)	4.52 (3.60,5.95)	0.369
Lymphocyte (X10^9/L)	1.50 (1.10,1.80)	1.40 (1.10,1.71)	1.50 (1.11,1.87)	1.45 (1.10,1.81)	0.518
Erythrocyte (X10^12/L)	4.30 ± 0.51	4.27 ± 0.50	4.24 ± 0.52	4.12 ± 0.52	0.008
Hemoglobin (g/L)	133 (120,144)	133 (123,142)	132 (120,144)	130 (116,139)	0.047
Platelet (X10^9/L)	219 (174,259)	206 (175,250)	203 (175,243)	201 (162,249)	0.183
Prothrombin Time (s)	13.3 (12.7,13.8)	13.2 (12.9,13.9)	13.4 (12.9,13.9)	13.4 (12.9,14.1)	0.523
INR	1.02 (0.97,1.07)	1.02 (0.97,1.08)	1.03 (0.97,1.08)	1.03 (0.97,1.10)	0.484
Fibrinogen (g/L)	3.55 (3.11,4.38)	3.71 (3.10,4.35)	3.61 (2.97,4.18)	3.67 (3.12,4.26)	0.740
**Comorbidities**					
Hypertension, n (%)	61 (35.9%)	61 (37.7%)	60 (36.6%)	70 (44.6%)	0.355
Diabetes mellitus, n (%)	18 (10.6%)	18 (11.1%)	17 (10.4%)	24 (15.3%)	0.480
Cardiovascular disease, n (%)	9 (5.3%)	10 (6.2%)	11 (6.7%)	12 (7.6%)	0.853
**Medication use**					
Atorvastatin therapy, n (%)	124 (72.9%)	118 (72.8%)	114 (69.5%)	122 (77.7%)	0.427
PAMBA therapy, n (%)	40 (23.5%)	45 (27.8%)	53 (32.3%)	51 (32.5%)	0.222
**Hematoma location**					0.367
Unilateral left, n (%)	68 (40.0%)	82 (50.6%)	75 (45.7%)	63 (40.1%)	
Unilateral right, n (%)	62 (36.5%)	48 (29.6%)	55 (33.5%)	51 (32.5%)	
Bilateral, n (%)	40 (23.5%)	32 (19.8%)	34 (20.7%)	43 (27.4%)	
**GOS at discharge**					0.042
5, n (%)	160 (94.1%)	149 (92.0%)	152 (92.7%)	132 (84.1%)	
4, n (%)	8 (4.7%)	10 (6.2%)	8 (4.9%)	22 (14%)	
3, n (%)	2 (1.2%)	3 (1.9%)	4 (2.4%)	3 (1.9%)	

**NOTE.** SBP, systolic blood pressure; DBP, diastolic blood pressure; Cr, creatinine; INR, international normalized ratio; PAMBA, para-aminomethylbenzoic acid; BUN, blood urea nitrogen; GOS, Glasgow Outcome Scale

### Association between the BUN concentrations and recurrence

Significant differences were observed between the recurrence and non-recurrence groups in BUN concentration quartiles of cases (*P* = 0.003). The proportion of cases in the lowest quartile (≤4.0 mmol/L) was dramatically low in the recurrence group (*P* = 0.027), whilst the proportion of cases in the highest quartile (> 6.4 mmol/L) was significantly high in the recurrence group (*P* = 0.012) (Table 4).

**Table 4:** BUN quartiles of patients

Variable	Recurrence (n = 96)	Non-Recurrence (n = 557)	χ^2^	*P*-value
BUN (mmol/L)			14.308	0.003
Quartile 1(≤4.0)	14 (14.6%)	156 (28.0%)	4.870	0.027
Quartile 2(> 4.0,≤4.9)	22 (22.9%)	140 (25.1%)	0.132	0.717
Quartile 3(> 4.9,≤6.4)	24 (25.0%)	140 (25.1%)	0.000	0.983
Quartile 4(> 6.4)	36 (37.5%)	121 (21.7%)	6.283	0.012

**Fig. 3:**
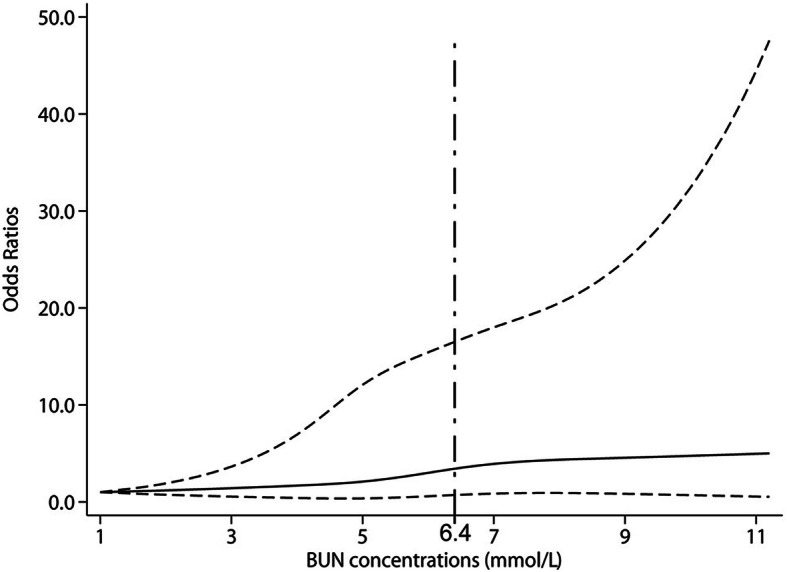
Association of BUN levels with risk of Recurrence. Dashed lines are 95% confidence intervals. Dotted line is where BUN concentration is 6.4 mmol/L. Odds ratios and 95% confidence intervals derived from restricted cubic spline regression. Odds ratios were estimated using logistic regression modeling, adjusting for the same variables as model 3 in Table 5

BUN, blood urea nitrogen;

In Table 5, with all cases taken as a whole, the condition that CSDH recurrence was interpreted as a dependent variable and the lowest quartile was interpreted as the reference was used for postoperative BUN level in the binary logistic regression models. The highest quartile of BUN concentration (> 6.4 mmol/L) was independently estimated as a risk factor of CSDH recurrence with an unadjusted OR of 3.315 (95%CI:1.711–6.423, *P* < 0.001). After adjusting for the confounders including sex, age, current alcohol drinking, current smoking, comorbidities (hypertension, diabetes mellitus, coronary heart disease), medicine (Atorvastatin and PAMBA), and laboratory investigation (platelet, fibrinogen, leukocyte, erythrocyte, hemoglobin, Cr), the highest quartile of BUN remained significantly and independently associated with CSDH recurrence (model 1: OR = 2.892, 95% CI:1.463–5.717, *P* = 0.002; model 2: OR = 2.939, 95% CI:1.480–5.836, *P* = 0.002; model 3: OR = 3.069, 95%CI:1.488–6.330, *P* = 0.002). There was no multicollinearity between the independent variables in model 3. Furthermore, restricted cubic spline regressions were used to explore the linear relationship between BUN concentration and the risk of CSDH recurrence (Fig. 3). Most importantly, it could be observed visually that the highest quartile had a significantly high OR value.

**Table 5:** Multivariate adjusted odds ratios for the association between BUN levels and Recurrence

	Quartile	OR^a^	95%CI	*P*-value
Unadjusted	Middle	1.751	0.863–3.554	0.121
	Higher	1.910	0.951–3.837	0.069
	Highest	3.315	1.711–6.423	< 0.001
Model 1^b^	Middle	1.590	0.778–3.252	0.204
	Higher	1.713	0.842–3.483	0.137
	Highest	2.892	1.463–5.717	0.002
Model 2^c^	Middle	1.612	0.788–3.299	0.191
	Higher	1.768	0.866–3.611	0.118
	Highest	2.939	1.480–5.836	0.002
Model 3^d^	Middle	1.612	0.780–3.333	0.197
	Higher	1.733	0.837–3.590	0.139
	Highest	3.069	1.488–6.330	0.002

OR, odds radio; CI confidence level; Cr, creatinine; Recurrence of postoperative CSDH

^a^ Reference OR (1.000) is the lowest quartile of BUN for Recurrence of CSDH

^b^ Model 1: adjusted for age, sex, current smoking, current alcohol drinking

^c^ Modal 2: adjusted for covariates from Model 1 and further adjusted for medical history (coronary heart disease, diabetes mellitus, hypertension) and medicine (Atorvastatin and PAMBA)

^d^ Modal 3: adjusted for covariates from Model 2 and further adjusted for Platelet, Fibrinogen, Leukocyte, Erythrocyte, Hemoglobin, Cr

## Discussion

To the best of our knowledge, this is the first study to investigate the association between BUN concentration and the recurrence of CSDH after evacuation. Our results indicated that high postoperative BUN concentration was related to the prevalence of CSDH recurrence within 3 months after the operation. Our findings hence suggest that the postoperative BUN concentration could be an available risk factor for CSDH recurrence.

BUN is a waste product of protein catabolism which is linked with poor prognosis and mortality in acute or chronic heart failure [12]. In addition, a study of 3355 AIS patients by You et al. observed that higher BUN had a 3.75-fold higher risk of in-hospital mortality [11]. In the present study, we found that patients with a relatively higher level of BUN were more likely to be moderately disabled at discharge. This was consistent with the previous studies that elevated BUN levels during hospitalization was related to a poor outcome. However, little data was found on the relationship between BUN and recurrence of CSDH. In this study, we observed that cases with a higher BUN level presented a trend toward a higher recurrence rate of CSDH. Furthermore, elevated BUN was associated with an enhanced risk of CSDH recurrence and cases with the highest postoperative BUN (> 6.4 mmol/L) seemed to have a 3.069-fold increase in danger of CSDH recurrence after adjusting for the potential confounders. The normal range of BUN level in the research hospital is 2.8–7.2 mmol/L which means the majority of patients presented with BUN level in the normal range. The BUN levels reflected not only the renal function but also other reactions that were mentioned below. In this study, the Cr level did not have an effect on the recurrence of CSDH which means there was no direct association between renal function and CSDH recurrence. The cutoff value of the postoperative BUN level in this study is 6.4 mmol/L and it indicated that the normal range of BUN level for intracranial lesion might be different from the systematic level. Also, elevated preoperative BUN levels showed an association with the recurrence of CSDH after logistic regression (See Supplemental Table, Additional file 1). Comparison between pre and post-operative BUN levels revealed that the BUN in the non-recurrence group significantly decreased after evacuation while it did not occur in the recurrence group, further confirming the relationship between BUN and CSDH recurrence.

The precise mechanisms underlying the association between elevated BUN and CSDH recurrence remained unclear. A potential explanation for this might be that a systemic increase in serum BUN induces intracranial multiple responses, which in turn can produce hematoma expansion and bring out CSDH recurrence. Some studies have revealed that the predictive value of BUN may be induced by its connection with other variables, such as protein intake, protein catabolism, nitrogen production and neurohormonal activation [12, 13]. On the other hand, CSDH was recently suggested to be formed through complex processes including angiogenesis, fibrinolysis and inflammation [14], and the recurrence of CSDH follows the same process as formation. There is strong evidence to show that vascular endothelial growth factor (VEGF) was related to the generation and steady increase of CSDH fluid volume [15–17] as well as the risk of CSDH recurrence [18]. Interestingly, Lin et al. observed that VEGF expression was positively correlated with BUN [19]. Hence, these discoveries might explain the relatively good correlation between elevated BUN and CSDH recurrence.

Compared with CSDH cases without recurrence, our result also showed that CSDH cases with recurrence had significantly higher age, which is consistent with the reports of previous studies [7, 20]. However, we found that the older cases were associated with higher BUN and the difference of patient age was not statistically significant after multivariate logistic regression analysis though age affects the recurrence of CSDH through the change of BUN.

Additionally, decreased fibrinogen level was found to be associated with the CSDH recurrence. In fact, fibrinogen has been proven to be a potentially available risk factor for postoperative intracranial bleeding [21, 22] considering that elevating serum fibrinogen was targeted as a valid therapy for intracranial hemorrhage [23]. Moreover, high levels of fibrinogen degradation products (FDPs) have been examined in CSDH fluid [24, 25] and fresh red cells were identified within CSDH fluid [24], suggesting that bleeding is an essential part of CSDH formation. The current study did not reveal a significant difference between postoperative atorvastatin use and CSDH recurrence. This finding runs contrary to existing studies [26, 27], hence, making the relationship between atorvastatin administration and CSDH recurrence puzzling.

There were a few limitations recognized in the present study. First, this study is a retrospective study and the selection bias is inevitable. Secondly, this is only a single-center clinical finding, which may impact the generalization of the results, thus further prospective research with plenty of patients might be required. Finally, considering that the role BUN plays in the CSDH recurrence is still confusing, more studies need to be pursued to further and better delineate CSDH formation in the future.

## Conclusion

The most obvious finding from this study was that elevated BUN at post-operation was independently associated with the recurrence of CSDH. The result suggested that a high level of postoperative BUN might serve as a risk factor of CSDH recurrence, so the determination of serum BUN after evacuation is important for patients with CSDH. Further prospective studies need to be undertaken to validate the causal relationship between BUN and CSDH recurrence.

## Supplementary Information

**Additional file 1.** Supplemental table of multivariate analyzed association between preoperative BUN and Recurrence.

**Additional file 2.** The complete data used and analysed during the current study.

## Data Availability

The datasets used and analyzed during the current study are available in supported Additional file 2 and the confidential patient data was erased.

## Abbreviations

CSDH: Chronic subdural hematoma
BUN: Blood Urea Nitrogen
BHI: Burr-hole irrigation
GCS: Glasgow coma score
CT: Computed tomography
MRI: Magnetic resonance imaging
AIS: Acute ischemic stroke
GOS: Glasgow Outcome Scale
ANOVA: Analysis of variance
OR: Odds ratio
CI: Confidence interval
Cr: Creatinine
PAMBA: Para-aminomethylbenzoic acid
SBP: Systolic blood pressure
DBP: Diastolic blood pressure
INR: International normalized ratio
VEGF: Vascular endothelial growth factor
FDPs: Fibrinogen degradation products
