# Supplementary material for: Elevated blood urea nitrogen is associated with recurrence of post-operative chronic subdural hematoma
Source: BMC Neurol. 2020 Nov 10;20:411. doi: 10.1186/s12883-020-01985-w (PMC7653870; doi:10.1186/s12883-020-01985-w)
Supplement: Supplementary file 1 — Additional file 1. Supplemental table of multivariate analyzed association between preoperative BUN and Recurrence. [file 12883_2020_1985_MOESM1_ESM.docx]

**Supplemental table**

Multivariate adjusted odds ratios for the association between preoperative BUN and Recurrence.

| Variables | OR^a^ | 95%CI | *P*-value |
| --- | --- | --- | --- |
| Preoperative BUN (mmol/L)^b^ |  |  | <0.001 |
| Quartile 2 | 7.266 | 1.597-32.698 | 0.010 |
| Quartile 3 | 10.845 | 2.407-48.854 | 0.002 |
| Quartile 4 | 49.595 | 11.624-211.609 | <0.001 |

BUN, blood urea nitrogen; OR, odds radio; CI confidence level.

^a^ Reference OR (1.000) is the lowest quartile of preoperative BUN for Recurrence of CSDH.

^b^ Adjusted for the same variables as model 2 in table 5.
